# Sacubitril Ameliorates Cardiac Fibrosis Through Inhibiting TRPM7 Channel

**DOI:** 10.3389/fcell.2021.760035

**Published:** 2021-10-29

**Authors:** Tian Jia, Xiaozhi Wang, Yiqun Tang, Wenying Yu, Chenhui Li, Shufang Cui, Juanjuan Zhu, Wei Meng, Chen Wang, Quanyi Wang

**Affiliations:** ^1^State Key Laboratory of Natural Medicines, Department of Life Sciences and Technology, China Pharmaceutical University, Nanjing, China; ^2^Department of Cardiology, The First Affiliated Hospital With Nanjing Medical University, Nanjing, China; ^3^Department of Clinical Pharmacy, School of Basic Medicine and Clinical Pharmacy, China Pharmaceutical University, Nanjing, China; ^4^State Key Laboratory of Natural Medicines, Department of Natural Medicinal Chemistry, China Pharmaceutical University, Nanjing, China

**Keywords:** sacubitril, cardiac fibrosis, PCD, TRPM7, Ca^2+^ influx

## Abstract

Heart failure caused by cardiac fibrosis has become a major challenge of public health worldwide. Cardiomyocyte programmed cell death (PCD) and activation of fibroblasts are crucial pathological features, both of which are associated with aberrant Ca^2+^ influx. Transient receptor potential cation channel subfamily M member 7 (TRPM7), the major Ca^2+^ permeable channel, plays a regulatory role in cardiac fibrosis. In this study, we sought to explore the mechanistic details for sacubitril, a component of sacubitril/valsartan, in treating cardiac fibrosis. We demonstrated that sacubitril/valsartan could effectively ameliorate cardiac dysfunction and reduce cardiac fibrosis induced by isoprotereno (ISO) *in vivo*. We further investigated the anti-fibrotic effect of sacubitril in fibroblasts. LBQ657, the metabolite of sacubitril, could significantly attenuate transforming growth factor-β 1 (TGF-β1) induced cardiac fibrosis by blocking TRPM7 channel, rather than suppressing its protein expression. In addition, LBQ657 reduced hypoxia-induced cardiomyocyte PCD *via* suppression of Ca^2+^ influx regulated by TRPM7. These findings suggested that sacubitril ameliorated cardiac fibrosis by acting on both fibroblasts and cardiomyocytes through inhibiting TRPM7 channel.

## Introduction

Heart failure (HF), as a secondary disease of various cardiovascular diseases (CVDs), is the leading cause of death worldwide. Its prevalence is projected to continuously increase, with aging of the population (Virani et al., 2021). The American Heart Association (AHA) data predicts that from 2012 to 2030, the prevalence of HF will increase by 46%, and the total percentage will increase from 2.42% in 2012 to 2.97% in 2030 (Virani et al., 2020). Current therapeutic strategies for patients mainly focus on stimulating contractility and reducing vasoconstriction (Burke et al., 2019). Cardiomyocyte loss through programmed cell death (PCD), including apoptosis, necrosis and autophagy-dependent cell death, play important roles in the pathogenesis of heart failure (Del Re and Amgalan, 2019). The progression of HF is commonly accompanied by development of cardiac fibrosis (von Lueder et al., 2015). Pathological stimuli such as oxidative stress and inflammation mediate the differentiation of cardiac fibroblasts (CFs) into myofibroblasts, which synthesize and deposit excessive extracellular matrix protein (ECM), initiating cardiac fibrosis (Tallquist, 2020; Wei et al., 2020). Fibrosis is a form of scarring that increases muscular tissue rigidity and decreases cardiac contractility. Currently, there is no effective treatment to block or reverse the development of fibrosis. The resulting abnormal cardiac conduction and stiffening of the ventricular walls severely damage the patients’ cardiac function, which may further cause abnormalities in other tissues and organs, and eventually death (Schimmel et al., 2020).

LCZ696 (sacubitril/valsartan) is recommended for the management of HF patients according to current guidelines (Yandrapalli et al., 2018). LCZ696 is the first-in-class Angiotensin Receptor Neprilysin Inhibitor (ARNi); it is a 1:1 combination of the angiotensin receptor blocker (ARB) valsartan and the neprilysin inhibitor (NEPi) prodrug sacubitril (Gu et al., 2010). Many experimental and clinical studies have suggested that the dual action of LCZ696 represents a major therapeutic advance in the management of HF (McMurray et al., 2014; Ishii et al., 2017; Yandrapalli et al., 2018; Burke et al., 2019). A potential direct anti-fibrotic role for valsartan has been established (von Lueder et al., 2015). NEPi sustains biologically active natriuretic peptides (NPs), that has been demonstrated to have beneficial effects on heart failure (Song et al., 2015). LBQ657 is a metabolite of sacubitril. It has the effect of inhibiting neprilysin, which degrades the natriuretic peptides and many other vasoactive peptides (Hubers and Brown, 2016). Effects of stand-alone LBQ657 on cardiac fibrosis have not been examined.

Ca^2+^ is a critical second messenger for the activation of cell signaling pathways involved in myocardial fibrosis and subsequent heart failure (Falcón et al., 2019). Abnormal endoplasmic reticulum and mitochondria cause intracellular free Ca^2+^ imbalance, which can trigger heart failure and atrial fibrillation, leading to morbidity and mortality of cardiac diseases (Dridi et al., 2020; Meyer and Doroudgar, 2020). Understanding the molecular basis of Ca^2+^-permeable channels is crucial for elucidating the mechanisms of abnormal cardiac function. Many studies have shown that transient receptor potential cation channel subfamily M (TRPM) channels participate in cardiac development and diseases (Wu et al., 2019; Simard and Magaud, 2020). TRPM member 7 (TRPM7) is a bifunctional protein with kinase structure and ion channel structure, which is responsible for Ca^2+^ influx (Yue et al., 2011; Duan et al., 2018). The important regulatory role of TRPM7 in cardiac hypertrophy, fibrosis and conduction disorders has been demonstrated sufficiently (Falcón et al., 2019). Our previous data confirmed that TRPM7 channel regulated hypoxia-induced myocardial fibrosis (Lu et al., 2017). Moreover, TRPM7 is broadly expressed and involved in many physiological and pathological processes such as cell proliferation and differentiation, cell death and transmembrane transport (Trapani and Wolf, 2020). Compelling evidences have demonstrated the pivotal roles of Ca^2+^ entry through TRPM7 in cardiac function and pathology (Du et al., 2010; Yu et al., 2014). Therefore, TRPM7 is becoming as a promising target to attenuate pathological cardiac fibrosis.

Herein reports direct evidence for anti-fibrotic function of sacubitril. We find sacubitril/valsartan has a better therapeutic effect on alleviating ISO-induced cardiac fibrosis and protecting against cardiac dysfunction *in vivo*, compared with valsartan alone. We further illustrate that LBQ657, the metabolite of sacubitril, ameliorates cardiac fibrosis through decreasing fibroblasts activation and reducing cardiomyocytes necrosis. The protective effect of LBQ657 against cardiac fibrosis is attributed to its impact on TRPM7-mediated Ca^2+^ entry by blocking TRPM7 channel. Taken together, the results of this study shed light on potential mechanism of sacubitril efficacy in heart fibrosis with reduced Ca^2+^ influx *via* acting on TRPM7 function.

## Materials and Methods

### Animal Experiment and Histological Examination

All animal experiments were carried out in accordance with the Guidelines of Animals Experiments from Ethical Committee for Animal Research of China Pharmaceutical University. Adult male Sprague–Dawley (SD) rats (weight 220–250 g) were purchased from the Model Animal Research Center of Nanjing University. The rats were raised under pathogen-free conditions, under the 12 light-dark cycle, 25 ± 2°C condition and free to water and food. Isoprotereno (ISO) (Sigma-Aldrich, United States) was used for cardiac fibrosis modeling. The rats were randomized into control group, ISO group, ISO + valsartan group and ISO + sacubitril/valsartan group. Valsartan was purchased from Changzhou Siyao Pharm, China; and sacubitril/valsartan was purchased from Novatis Pharma, Schweiz AG. Rats in control group were subcutaneously injected with saline, while the other three groups were subcutaneously injected with ISO (7.5 mg/kg/day) for 14 days. From the 6th day to the 14th day, the rats in control group and ISO group were given saline by gavage (0.01 mL/g body weight), the rats in ISO + valsartan group were given valsartan by gavage (0.01 mL/g body weight, dissolved in 0.5% CMC-Na solution), and the rats in ISO + sacubitril/valsartan group were given sacubitril valsartan by gavage (0.01 mL/g body weight, dissolved in 0.5% CMC-Na solution). On the 15th day, rats were anesthetized with 2% isoflurane and kept on a 37°C heated platform. The chest hairs were removed using depilatory cream and acoustic coupling gel was applied to the thorax, then echocardiogram tests were performed using small animal ultrasound imaging system vevo3000 (VisualSonics, Canada). The cardiac function related indicators, including cardiac output, ejection fraction, fractional shortening, and stroke volume, were analyzed through the echocardiogram results. Then all rats were sacrificed and heart tissues were taken for follow-up analysis. The left ventricular apexes were resected and divided into two parts: one part was fixed in 10% formalin solution and then subjected to Masson trichrome staining to observe the histological changes, while the other part was frozen at −80°C for subsequent detection of mRNA and protein.

### Cell Culture

Mouse embryonic fibroblast (MEF) and rat embryonic cardiomyocyte cell line H_9_C_2_ were cultured in DMEM (Gibco, United States) containing 10% FBS (Gibco, United States), 100 U/mL penicillin and 100 μg/mL streptomycin (Gibco, United States). Cells were digested with 0.25% trypsin-EDTA (Gibco, United States) and subcultured for subsequent experiments when grew to 90% confluence.

### Small Interfering RNA Transfection

Si-TRPM7 and si-NC were synthesized by GenePharm (Shanghai, China). MEF were seeded in 12-well cell culture plate (NEST, China) and transfected with small interfering RNAs (siRNAs) using Lipofectamine^TM^ 3000 Transfection Reagent (ThermoFisher, United States) in serum-free Opti-MEM medium (Gibco, United States). The cells were transfected for 8 h and then cultured in DMEM containing 10% FBS, 100 U/mL penicillin and 100 μg/mL streptomycin. The sequences of si-TRPM7 are as following:

si-TRPM7 sense 5′-GCGCUUUCCUUAUCCUCUUTT-3′;
si-TRPM7 antisense 5′-AAGAGGAUAAGGAAAGCGCTT-3′.

### Quantitative Reverse Transcription-Polymerase Chain Reaction

Total RNA of cells or tissues was extracted with Trizol (Invitrogen, United States) according to the manufacturer’s instructions, then reverse transcription was performed using a reverse transcription kit (Vazyme, China). The quantification of gene transcripts was performed by real-time PCR using SYBR Green PCR mix (Vazyme, China). All values were normalized to the level of *18S* mRNA. The primers used were listed below:

*18S* sense CGAACGTCTGCCCTATCAACT;
*18S* antisense CAGACTTGCCCTCCAATGGATCCTCGTT;
Rat-*Mmp9* sense CAGAGCGTTACTCGCTTGGA;
Rat-*Mmp9* antisense GGTTGTGGAAACTCACACGC;
Mouse-*Mmp9* sense GTCCAGACCAAGGGTACAGC;
Mouse-*Mmp9* antisense ATACAGCGGGTACATGAGCG;
Rat-α*-Sma* sense ACCATCGGGAATGAACGCTT;
Rat-α*-Sma* antisense CTGTCAGCAATGCCTGGGTA;
Mouse-α*-Sma* sense AGCCATCTTTCATTGGGATGG;
Mouse-α*-Sma* antisense CCCCTGACAGGACGTTGTTA;
Rat-*Trpm7* sense TGTTGCCGGATTGGTTACGA;
Rat-*Trpm7* antisense CTCGTGGAGGTACAGGAACG.

### Western Blot

Cell protein was extracted by sonication and tissue protein was extracted by grinding. After centrifugation at 12,000 rpm for 15 min, the supernatant was collected for subsequent western blot analysis. Protein samples were boiled for 5 min, electrophoresed in SDS polyacrylamide gel, and then transferred onto PVDF membranes (Bio-Rad, United States). The blots were blocked with 5% skimmed milk in Tris-buffered saline solution-Tween 0.1% (TBST) (Sigma-Aldrich, United States) for 2 h at room temperature and probed with primary antibodies overnight at 4°C. The blots were washed four times with TBST for 8 min each time and incubated for 1 h at room temperature with the HRP-conjugated secondary antibodies (dilution 1:10,000; Sigma-Aldrich, United States), then developed with chemiluminescence (Vazyme, China). MMP9 antibody (10375-2-AP) was purchased from Proteintech, China, dilution 1:1,000; α-SMA (ab32575) and MLKL (ab243142) antibodies were purchased from Abcam, United States, dilution 1:1,000; TRPM7 antibody (BM5443) was purchased from Boster, China, dilution 1:200; LC3B (3868S), caspase3 (14220S) and cleaved caspase3 (9664S) were purchased from Cell Signaling Technology, United States, dilution 1:1,000; RIPK1 (AF7877), RIPK3 (AF7942) and phospho-MLKL (Ser358) (AF7420) antibodies were purchased from Affinity, China, dilution 1:1,000. The densitometry of protein bands was quantified using ImageJ software. All values were normalized to the level of β-actin.

### Molecular Docking of TRPM7 Ligands

The crystal structure of TRPM7 (PDB ID: 5ZX5) (Duan et al., 2018) was retrieved from the RCSB Protein Data Bank. The protein structure was prepared for Glide docking calculations using the Protein Preparation Wizard utility. Water molecules were removed and the structure was treated and prepared by employing a protein preparation panel in the Schrödinger enterprise to magnify H-bond interactions. Structures of investigated compound (NEPi and Diprotic acid ARB) were built recruiting MAESTRO (Friesner et al., 2004) build panel and subsequently energy minimization by LigPrep module employing the OPLS2005 forcefield. Ionizable compounds were converted to their most probable charged forms at pH 7.0 ± 2.0 and explicit hydrogen atoms were added. The grid file for TRPM7 was generated by the Glide Grid Generation wizard in Schrödinger determining the co-crystallized ligands as the center point. Van der Waals scaling factor of the non-polar atoms was adjusted to 1.0 to enhance flexibility. The other parameters were fixed as defaults. The top conformations of investigated compounds were ranked by the corresponding values of docking score.

### Isolation of Neonatal Rat Cardiac Fibroblasts

Hearts from the neonatal rats (1–3 days) were rapidly removed and washed in D-hanks solution and minced into 1 mm^3^ pieces, then shake in 0.1% trypsin and 0.1% collagenase type II for several times to digest. The fully digested cell suspension was centrifuged and resuspended in Dulbecco’s modified eagle’s medium (DMEM) (Life Technologies, United States) containing 10% FBS, 100 U/mL penicillin and 100 μg/mL streptomycin, and incubated in a humidified atmosphere of 5% CO_2_ at 37°C for 2 h. After removing non-adhered cells, the attached cells were cultured and inherited, the second or third generations were used in our experiments.

### Whole Cell Patch Clamp

The third passage of neonatal rat cardiac fibroblasts was used in our experiments. TRPM7-like current was recorded by whole cell patch clamp at room temperature. The pipette solution contained (in mM) CsCl 145, NaCl 8, HEPES 10, EGTA 10, and CsOH was used to adjust to pH7.2. The bath solution contained (in mM) NaCl 145, KCl 5, HEPES 10, glucose10 and CaCl_2_ 2, and NaOH was used to adjust to pH7.4. With a holding potential at 0 mV, TRPM7 current was recorded under the voltage stimulation from −100 mV to + 120 mV in 400 ms.

### Hypoxia Treatment Procedure

H_9_C_2_ were divided into normoxic group, hypoxia group, hypoxia + BAPTA-AM group and hypoxia + LBQ657 group. BAPTA-AM and LBQ657 were purchased from MedChemExpress, China and Sigma-Aldrich, United States, respectively. Cells were seeded in 6-well cell culture plate (NEST, China) at a density of 5 × 10^5^ per well. 8 h later, cells were treated with BAPTA-AM or LBQ657 for 8 h and then moved to a hypoxia environment by incubating into three-gas incubator (93% N_2_, 5% CO_2_ and 2% O_2_) for 24 h to achieve, while the control groups were kept in normoxic incubator. All cell pellets were collected and total protein were extracted for western blot test.

### Confocal Microscopy

H_9_C_2_ were loaded with the Ca^2+^ fluorescent dye, Fluo-4-AM (Invitrogen, United States) at 37°C for 1 h incubation, then washed by PBS solution and incubated at 37°C for 30 min. Cells were fixed by 4% paraformaldehyde and screened in 488 nm excitation light using Laser scanning confocal microscope LSM800 (Zeiss, Germany). The fluorescence ratio was analyzed using Image J software.

### Statistical Analysis

Statistical analysis was performed using GraphPad Prism 6.0. The results were expressed as mean ± SD for experiments conducted at least in triplicates. Unpaired *t* test was used for comparison between two groups, and One-way ANOVA was used for three or more groups, and *P* < 0.05 was considered to be significantly different.

## Results

### Sacubitril/Valsartan Effectively Protected Cardiac Function and Ameliorated Cardiac Fibrosis Induced by ISO in Rats

**FIGURE 1:**
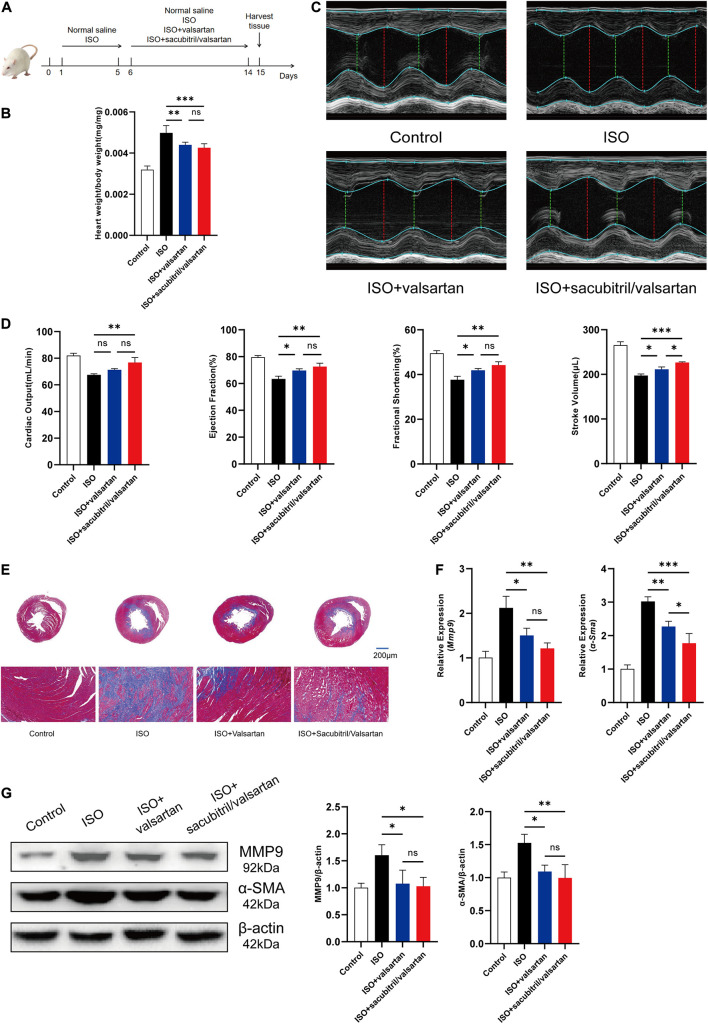
Sacubitril/valsartan effectively protected cardiac function and ameliorated cardiac fibrosis induced by ISO in rats. **(A)** The flow chart of the cardiac fibrosis model and drug treatment in animal experiment. **(B)** Heart to body weight ratio of rats in the control group, the ISO group, the valsartan group and the sacubitril/valsartan group. **(C)** On the 15th day of the experiment, the heart function of rats was measured by echocardiography**. (D)** Cardiac output, ejection fraction, fractional shortening and stroke volume were analyzed and calculated according to the results of echocardiography. **(E)** Masson trichrome staining of heart tissue sections of rats. Scale bar = 200 μm. **(F)** mRNA expression levels of *Mmp9* and α*-Sma* in the left ventricular apexes of rats. **(G)** Protein levels of MMP9 and α-SMA in the left ventricular apexes of rats. The data presented are mean ± SD. *n* = 5/group. *18S* RNA was used as the internal reference gene and β-actin was used as the internal reference protein for normalization and statistical analysis. ^∗∗∗^*P* < 0.001, ^∗∗^*P* < 0.01, and ^∗^*P* < 0.05.

The rats were subcutaneous injected ISO to induce cardiac fibrosis and treated with normal saline, valsartan or sacubitril/valsartan, respectively (Figure 1A). Valsartan, a drug used for clinical treatment of heart failure, was used as positive control (Suematsu et al., 2016). The valsartan group and the sacubitril/valsartan group had lower heart weight to body weight ratio (Figure 1B). Echocardiogram showed that the cardiac output, ejection fraction, fractional shortening and stroke volume of ISO-induced rats were significantly increased after valsartan and sacubitril/valsartan treatment (Figures 1C,D). Especially, sacubitril/valsartan ameliorated stroke volume more efficiently than valsartan. Additionally, the valsartan group and the sacubitril/valsartan group had smaller area of fibrosis (Figure 1E) compared to non-treatment control group. These results indicate that sacubitril/valsartan effectively protected cardiac dysfunction in ISO-stimulated rats (*P* < 0.01 compared with control group).

Matrix metalloproteinase 9 (MMP9) is a biomarker associated with collagen degradation to cause heart failure (Zile et al., 2019). Alpha-smooth muscle actin (α-SMA) is an activation marker of fibrotic pathways in fibroblast differentiation (Jing et al., 2017). Accordingly, quantitative reverse transcription-polymerase chain reaction (qRT-PCR) was used to analyze the expression of these cardiac fibrosis related genes in the left ventricular apexes. We found that ISO induced a notable increase in *Mmp9* and α*-Sma* mRNA expression levels, while sacubitril/valsartan or valsartan treatment diminished the effect (Figure 1F). The protein expression levels of MMP9 and α-SMA detected by western blotting analysis showed a similar trend (Figure 1G). Moreover, sacubitril/valsartan significantly decreased α*-Sma* mRNA expression compared with valsartan. Taken together, the above results suggest that sacubitril/valsartan effectively ameliorated ISO induced cardiac fibrosis and improved cardiac function *in vivo*, superior to valsartan therapy alone. This indicates a potential anti-fibrotic function of sacubitril.

### LBQ657 Significantly Extenuated TGF-β1 Induced Cardiac Fibroblast Activation *in vitro*

**FIGURE 2:**
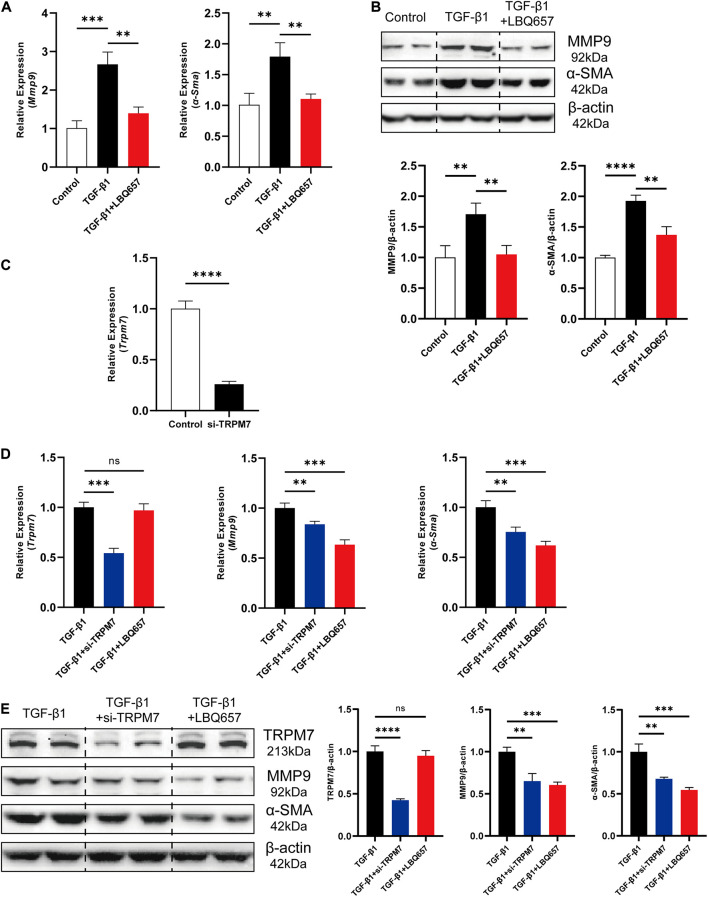
LBQ657 significantly extenuated TGF-β1 induced cardiac fibroblast activation in MEF. **(A)** mRNA expression of *Mmp9* and α*-Sma* in control MEF, MEF treated with TGF-β1 and MEF treated with LBQ657 + TGF-β1. **(B)** Protein levels of MMP9 and α-SMA in the rats. **(C)** The efficiency of the siRNA used to reduce the mRNA expression of TRPM7 in MEF. **(D)** mRNA expression of *Trpm7*, *Mmp9* and α*-Sma* in MEF treated with TGF-β1, MEF transfected with si-TRPM7 and treated with TGF-β1, MEF treated with LBQ657 + TGF-β1. **(E)** Protein levels of TRPM7, MMP9 and α-SMA in the rats. The data presented are mean ± SD. Each group contained the results of three independent repeated trials. *18S* RNA was used as the internal reference gene and β-actin was used as the internal reference protein for normalization and statistical analysis. ^∗∗∗∗^*P* < 0.0001, ^∗∗∗^*P* < 0.001, and ^∗∗^*P* < 0.01.

Transforming growth factor-β 1 (TGF-β1) is considered as a typical stimulus to mediate the differentiation of cardiac fibroblasts into myofibroblasts to acquire a fibrotic phenotype (Porter and Turner, 2009). To further examine the effect of sacubitril on cardiac fibrosis, we used TGF-β1 (10 ng/mL) to stimulate MEF for 24 h to induce a profibrotic phenotype. LBQ657, the metabolite of sacubitril, was used to treat MEF cells at a concentration of 50 μM prior to TGF-β1 stimulation. We found both the mRNA and protein expression of MMP9 and α-SMA was dramatically up-regulated by TGF-β1 stimulation, but this was prevented by LBQ657 pre-treatment (*P* < 0.01 comparison with the TGF-β1 group), indicating LBQ657 potently inhibited fibroblast activation *in vitro* (Figures 2A,B).

Because the previous reports suggested a close relationship between TRPM7 and cardiac fibrosis (Du et al., 2010; Wei et al., 2020), we further investigated the changes of TRPM7 expression in TGF-β1 induced cardiac fibrosis. We designed and selected a pair of TRPM7 siRNA with the best interference effect for follow-up experiments. The TRPM7 mRNA levels were reduced by 74% (Figure 2C). MEF were subjected to si-TRPM7 transfection and LBQ657 (50 μM) treatment, respectively, before TGF-β1 stimulation. The qRT-PCR and western blotting analysis indicated that si-TRPM7 suppressed the expression of TRPM7, but LBQ657 pre-treatment did not show discernible effects on TRPM7 protein expression in TGF-β1 treated MEF (Figures 2E,F). Meanwhile, both si-TRPM7 and LBQ657 reduced the expression of MMP9 and α-SMA induced by TGF-β1 (Figures 2D,E). These results suggest that LBQ657 attenuated TGF-β1-induced cardiac fibroblast activation without reducing the expression levels of TRPM7.

### LBQ657 Protected Against Fibrosis by Blocking TRPM7 Channel

**FIGURE 3:**
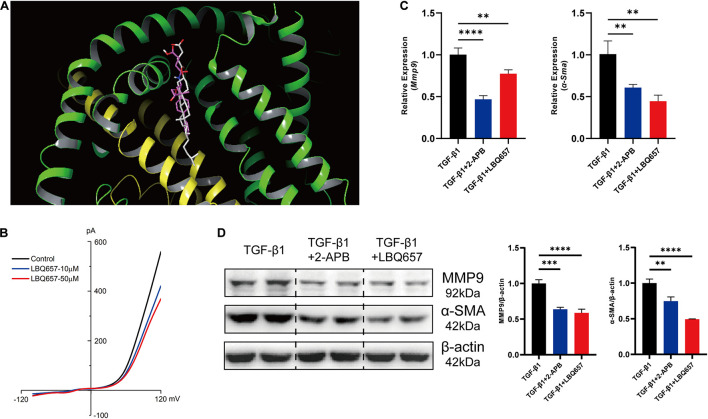
LBQ657 protected against fibrosis by blocking TRPM7 channel. **(A)** The virtual binding mode of NEPi and natural cholesterol hemisuccinateon ligand (as control) on TRPM7. NEPi (red) and cholesterol hemisuccinate (gray) are shown as a stick model. **(B)** Current amplitude of TRPM7-like current recorded in neonatal rat cardiac fibroblasts in control cell (black), LBQ657-10 μM treated cell (blue) and LBQ657-50 μM treated cell (red). **(C)** mRNA expression of *Mmp9* and α*-Sma* in MEF treated with TGF-β1, MEF treated with 2-APB + TGF-β1, and MEF treated with LBQ657 + TGF-β1. **(D)** Protein levels of MMP9 and α-SMA in the rats. The data presented are mean ± SD. Each group contained the results of three independent repeated trials. *18S* RNA was used as the internal reference gene and β-actin was used as the internal reference protein for normalization and statistical analysis. ^∗∗∗∗^*P* < 0.0001, ^∗∗∗^*P* < 0.001, and ^∗∗^*P* < 0.01.

The inhibition of cardiac fibroblast activation by LBQ657 was not associated with reduced TRPM7 mRNA and protein levels, ruling out the possibility that LBQ657 functions by regulating TRPM7 channel current. Molecular docking was used to determine the interaction between TRPM7 (PDB ID: 5ZX5) and sacubitril/valsartan. Compound NEPi and Diprotic acid ARB of sacubitril/valsartan docked to the active site of TRPM7 with a free energy of −6.20 kcal/mol and −10.12 kcal/mol, respectively. This indicated that both sacubitril and valsartan docked to TRPM7 protein. The docking structure suggested that LBQ657, as the NEPi, occupied a similar position in TRPM7 compared with natural cholesterol hemisuccinateon ligand (Figure 3A).

Aimed to validate the blocking of TRPM7 channel by LBQ657, TRPM7-like current was recorded by whole cell patch-clamp in neonatal rat cardiac fibroblasts. We observed that the current of TRPM7 channel was decreased by LBQ657 treatment in a clear dose-dependent manner (Figure 3B), strongly implying its direct influence on the channel function. And then we used 2-aminoethoxydiphenyl borate (2-APB), an inhibitor of TRPM7 channel, as a positive control to explore the anti-fibrotic effect of LBQ657 (Chokshi et al., 2012). As shown in Figures 3C,D, 2-APB (100 μM) indeed caused dramatic reduction of the MMP9 and α-SMA expression by ∼50%, so did LBQ657, in TGF-β1 stimulated MEF cells. We concluded from this set of experiments that the LBQ657 extenuated cardiac fibroblast activation through inhibiting the function of TRPM7 channel, rather than suppressing the expression of TRPM7.

### LBQ657 Reduced Hypoxia-Induced Necrosis by Inhibiting TRPM7-Mediated Ca^2+^ Influx in Cardiomyocytes

**FIGURE 4:**
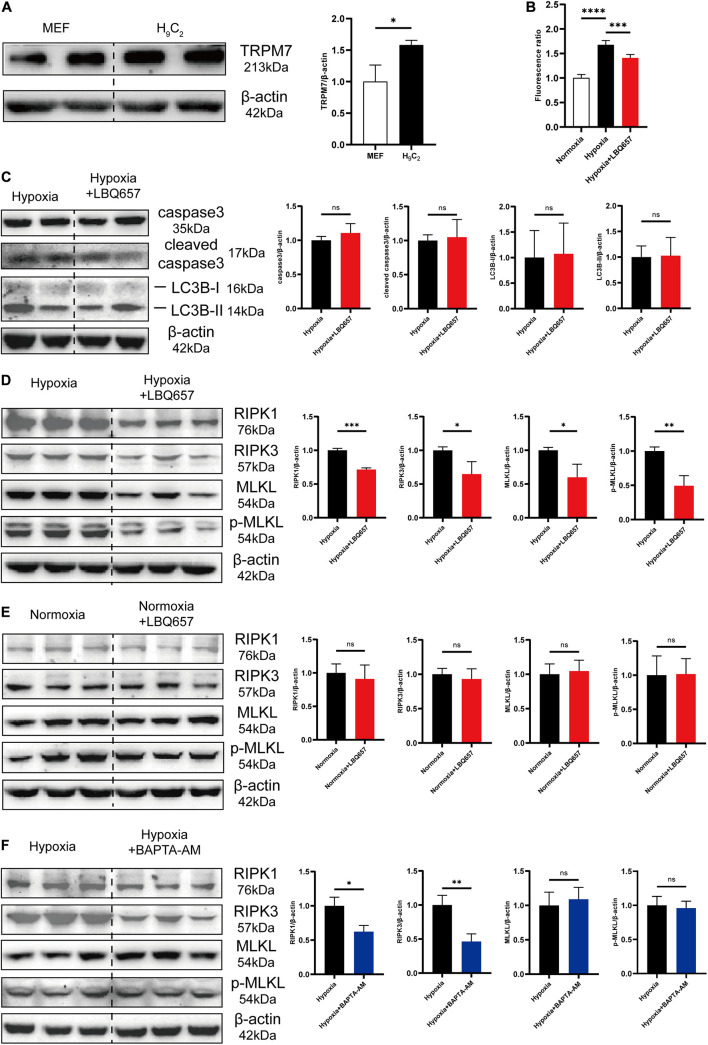
LBQ657 reduced hypoxia-induced necrosis by inhibiting TRPM7-mediated Ca^2+^ influx in cardiomyocytes. **(A)** Comparison of TRPM7 protein levels in MEF and H_9_C_2_ cells. **(B)** Fluorescence intensity of Fluo-4-AM fluorescent dye in 488 nm excitation light of normoxic H_9_C_2_, hypoxic H_9_C_2_ and hypoxic H_9_C_2_ pre-treated with LBQ657. **(C)** Protein levels of caspase3, cleaved caspase3, LC3B-I and LC3B-II in hypoxic H_9_C_2_ and hypoxic H_9_C_2_ pre-treated with LBQ657. **(D)** Protein levels of RIPK1/RIPK3/MLKL/p-MLKL in hypoxic H_9_C_2_ and hypoxic H_9_C_2_ pre-treated with LBQ657. **(E)** Protein levels of RIPK1/RIPK3/MLKL/p-MLKL in normoxic H_9_C_2_ and normoxic H_9_C_2_ treated with LBQ657. **(F)** Protein levels of RIPK1/RIPK3/MLKL/p-MLKL in hypoxic H_9_C_2_ and hypoxic H_9_C_2_ pre-treated with BAPTA-AM. The data presented are mean ± SD. Each group contained the results of three independent repeated trials. β-actin was used as the internal reference protein for normalization and statistical analysis. ^∗∗∗∗^*P* < 0.0001, ^∗∗∗^*P* < 0.001, ^∗∗^*P* < 0.01, and ^∗^*P* < 0.05.

The causal importance of hypoxia in cardiac fibrosis has been strongly supported by literature. Hypoxia occurs in myocardial infarction and causes large-scale loss of cardiomyocytes, which is the most common trigger of cardiac fibrosis. Cardiomyocyte death induces inflammatory responses, promoting the activation of fibroblasts into myofibroblasts, leading to cardiac fibrosis (Kong et al., 2014; Kumar and Choi, 2015). TRPM7 channel is mainly concentrated in myocardium during embryonic development and is indispensable for cardiac automaticity of cardiomyocytes (Sah et al., 2013). Therefore, we detected that TRPM7 was abundantly expressed in cardiomyocytes, approximately 1.5-fold greater than that in fibroblasts (Figure 4A). And then we measured the intracellular calcium concentration in H_9_C_2_ under hypoxia stimulation. The Figure 4B showed that Ca^2+^ influx was dramatically increased by hypoxia, but significantly deceased after LBQ657 treatment. These results demonstrated that LBQ657 inhibited Ca^2+^ entry though TRPM7 channel in cardiomyocytes.

While some studies had previously shown that activation of TRPM7 channels and produced Ca^2+^ overload significantly contributes to cell death (Wei et al., 2007; Cai et al., 2014), it was still to be determined whether LBQ657 attenuates cardiomyocyte PCD by TRPM7 mediated Ca^2+^ influx. We first studied the effect of LBQ657 on hypoxia-induced cardiomyocyte PCD. As shown in Figure 4C, there were no significant changes on the protein expression level of caspase3, cleaved caspase3, LC3B-I and LC3B-II in hypoxia-stimulated H_9_C_2_ pre-treated with LBQ657, suggesting that LBQ657 was incapable of regulating cardiomyocyte apoptosis or autophagy in hypoxic environment. The investigation of cell necrosis related proteins RIPK1/RIPK3/MLKL/p-MLKL expression in hypoxia-induced H_9_C_2_ under LBQ657 pre-treatment indicated the expression level of detected proteins declined to 70% or less compared with that in the hypoxia group (Figure 4D). We further found LBQ657 did not cause obvious changes in the expression level of RIPK1/RIPK3/MLKL/p-MLKL in normoxia cardiomyocytes (Figure 4E). Next, BAPTA-AM (5 μM) was used to chelate the intracellular calcium and caused down-regulated expression of RIPK1/RIPK3 in hypoxia-treated cardiomyocytes, but MLKL and p-MLKL protein expression were not notably altered (Figure 4F). These results suggested that the reduction of cardiomyocyte necrosis was closely associated with calcium influx. The cardiomyocyte necrosis was significantly reduced accompanied with decrease of TRPM7-mediated Ca^2+^ influx. Based on the above results, we conclude that LBQ657 could ameliorated cardiomyocyte necrosis induced by hypoxia *via* inhibiting TRPM7-mediated Ca^2+^ influx.

## Discussion

**FIGURE 5:**
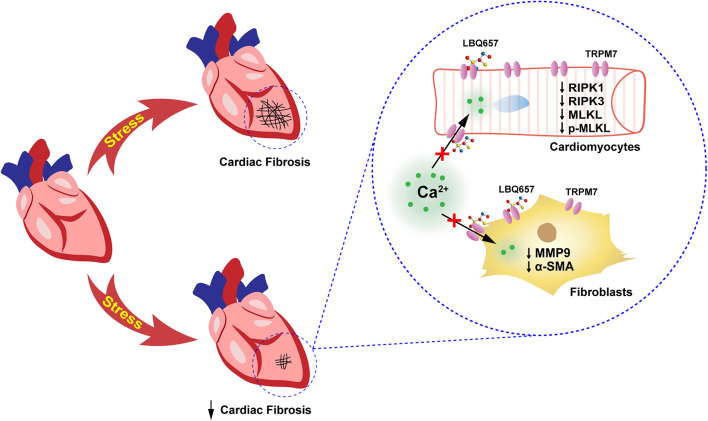
Schematic showing that sacubitril ameliorates cardiac fibrosis through inhibiting TRPM7-mediated Ca^2+^ influx in cardiac fibroblasts and cardiomyocytes. This study demonstrated that sacubitril metabolite LBQ657 could relieve the fibrotic response of fibroblasts and reduce cardiomyocyte PCD by blocking TRPM7 channel, thereby ameliorating cardiac fibrosis.

As one of the leading causes of deaths, HF places a tremendous burden to the healthcare systems of countries worldwide. LCZ696 (sacubitril/valsartan) represents a promising therapeutic drug in the treatment of CVD, particularly HF. LCZ696 can reduce cardiac fibrosis and hypertrophy to lower the risk of cardiovascular events in HF and attenuate cardiac remodeling and dysfunction after myocardial infarction (MI) (von Lueder et al., 2015; Ishii et al., 2017). In addition, LCZ696 reduces fibrosis and inflammation in chronic kidney disease (Jing et al., 2017), ameliorates cardiac function in diabetic mice (Suematsu et al., 2016), and improves clinical prognosis in patients with heart failure with reduced ejection fraction (HFrEF) (Cunningham et al., 2020). Since its development and commercialization, many studies have assessed its pathophysiological effects, as well as pharmacokinetics and pharmacodynamics (Ayalasomayajula et al., 2017; Hsiao et al., 2018; Zile et al., 2019). However, relatively few studies have deciphered the underlying mechanism involved in its anti-fibrotic effect. Our present study has shown that LBQ657 (active form of sacubitril) protected against cardiac fibrosis, probably due to the suppression of TRPM7 function in both cardiac fibroblasts and cardiomyocytes (Figure 5).

Published reports have shown that valsartan, the angiotensin receptor blocker component of LCZ696, inhibited the renin-angiotensin-aldosterone system to exert anti-fibrotic function (Lin et al., 2016). In addition, accumulating evidence also suggested that LCZ696 was more effective than valsartan alone in inhibiting fibrosis (Suematsu et al., 2016; Jing et al., 2017). However, the mechanism of anti-fibrosis by LBQ657, the neprilysin inhibitor component of LCZ696, is elusive. von Lueder et al. (2015) suggested that LBQ657 inhibited post-MI hypertrophy but not fibrosis. While Yandrapalli et al. (2017) revealed that augmenting the NPS inhibited the progression of HF. In this study, the anti-fibrotic effect of LBQ657 leads to the attenuation of ISO-dependent fibrosis, the inhibition of TGF-β1-induced fibroblast activation, and reduction of hypoxia-induced cardiomyocyte PCD. Based on above observations, we speculate that LBQ657 synergizes with valsartan in inhibiting cardiac fibrosis.

The “chanzyme” TRPM7 contains both an ion channel and an α-kinase, which might be the molecular basis of the major divalent cation permeable channel in cardiac function and pathology (Falcón et al., 2019). In human atrial fibrillation, Ca^2+^ entry through TRPM7 channel plays a pivotal role in TGF-β1-elicited fibrogenesis in atrial fibroblasts (Du et al., 2010). While former attention was made to the altered expression of TRPM7 and the development of cardiac fibrosis (Xu et al., 2015), our current study indicated that LBQ657 administration improved cardiac function and attenuated the molecular markers (MMP9 and α-SMA) of fibrosis in fibroblasts by suppressing TRPM7 channel Ca^2+^ influx rather than by decreasing TRPM7 mRNA and protein levels. Ca^2+^ permeation may also account for the mechanism of TRPM7’s action in cardiac dysfunction.

The term programmed cell death is applied broadly to refer to a number of cell death programs, including apoptosis, regulated necrosis, ferroptosis, pyroptosis, parthanatos, entotic cell death, lysosome-dependent cell death, autophagy-dependent cell death, and immunogenic cell death (Del Re and Amgalan, 2019; Bedoui et al., 2020; Khan et al., 2021). Autophagy is a process of cellular self-degradation for keeping the intracellular homeostasis (Bhardwaj et al., 2020). Although increased autophagy protects against various pathological factors in cardiomyocytes, the mistakenly degradation of essential cellular components by excessive autophagy exacerbates myocardial injury (Luo et al., 2021). Apoptosis is an intrinsic cell death program caused by diverse factors. There is a significant association between improved cardiac function and prevention of cardiomyocyte apoptosis in human HF (Li et al., 2021). The role for regulated forms of cardiomyocyte necrosis is critical in both myocardial infarction and heart failure. Cardiac Ca^2+^ overload has been proved to induce cardiomyocyte necrosis, heart failure, and premature death (Del Re and Amgalan, 2019). Previously, a systems biology approach has revealed that sacubitril attenuated cardiomyocyte cell death by inhibiting PTEN (Iborra-Egea et al., 2017). TRPM7-mediated Ca^2+^ influx was identified as a crucial mechanism of tumor necrosis factor (TNF)-induced necroptosis (Cai et al., 2014) and neuronal death during transient brain ischemia (Wei et al., 2007). Indeed, we show here that regulation of TRPM7-mediated Ca^2+^ influx by LBQ657 contributed to the reduction of hypoxia-induced cardiomyocyte necrosis, rather than apoptosis or autophagy. The protective effect of LBQ657 against cardiovascular fibrosis is demonstrated to be achieved at least partially through Ca^2+^-sensitive PCD.

Taken together, our study sheds a new light in understanding the mechanisms by which sacubitril attenuates cardiac fibrosis, and has important guiding significance for clinical treatment. LBQ657, an active metabolite of sacubitril, inhibits cardiac fibrosis by acting on both cardiac fibroblasts and cardiomyocytes; and TRPM7 channel is the target of LBQ657 in these cells. As a major advance in the treatment of CVDs, sacubitril/valsartan should be evaluated as a direct anti-fibrotic therapy, better than valsartan. Importantly, our work identifies LBQ657 is a pharmacological compound that inhibits the TRPM7 channel function for the first time. This will open a door to develop LBQ657 as a new modulator to unravel TRPM7 channel vs. kinase function in cellular physiology and pathophysiology, and as a new therapy for various cardiovascular diseases.

## Data Availability Statement

The original contributions presented in the study are included in the article/supplementary material, further inquiries can be directed to the corresponding author/s.

## Ethics Statement

The animal study was reviewed and approved by Ethical Committee for Animal Research of China Pharmaceutical University.

## Author Contributions

TJ, XW, CL, SC, JZ, and WM performed the molecular and animal experiments. YT performed the patch clamp. WY performed the compounds and protein docking analysis. QW and CW designed and supervised the whole project. All authors contributed to manuscript revision, read, and approved the submitted version.

## Conflict of Interest

The authors declare that the research was conducted in the absence of any commercial or financial relationships that could be construed as a potential conflict of interest.

## Publisher’s Note

All claims expressed in this article are solely those of the authors and do not necessarily represent those of their affiliated organizations, or those of the publisher, the editors and the reviewers. Any product that may be evaluated in this article, or claim that may be made by its manufacturer, is not guaranteed or endorsed by the publisher.
